# STUMP un“stumped”: anti-tumor response to anaplastic lymphoma kinase (ALK) inhibitor based targeted therapy in uterine inflammatory myofibroblastic tumor with myxoid features harboring *DCTN1-ALK* fusion

**DOI:** 10.1186/s13045-015-0160-2

**Published:** 2015-06-11

**Authors:** Vivek Subbiah, Caitlin McMahon, Shreyaskumar Patel, Ralph Zinner, Elvio G Silva, Julia A. Elvin, Ishwaria M. Subbiah, Chimela Ohaji, Dhakshina Moorthy Ganeshan, Deepa Anand, Charles F. Levenback, Jenny Berry, Tim Brennan, Juliann Chmielecki, Zachary R. Chalmers, John Mayfield, Vincent A. Miller, Philip J. Stephens, Jeffrey S. Ross, Siraj M. Ali

**Affiliations:** Division of Cancer Medicine, Department of Investigational Cancer Therapeutic (Phase I Clinical Trials Program), The University of Texas MD Anderson Cancer Center, 1515 Holcombe Blvd, FC8.3038, Box 0455, Houston, TX 77030 USA; Foundation Medicine, Inc, Cambridge, MA 02141 USA; Division of Cancer Medicine, Department of Sarcoma Medical Oncology, The University of Texas MD Anderson Cancer Center, 1515 Holcombe Blvd, Houston, TX 77030 USA; Division of Diagnostic Pathology, The University of Texas MD Anderson Cancer Center, 1515 Holcombe Blvd, Houston, TX 77030 USA; Division of Diagnostic Imaging and Radiology, The University of Texas MD Anderson Cancer Center, 1515 Holcombe Blvd, Houston, TX 77030 USA; Division of Surgery, Department of Gynecologic Oncology and Reproductive Medicine, The University of Texas MD Anderson Cancer Center, Houston, TX USA

**Keywords:** ALK, Sarcoma, Inflammatory myofibroblastic tumor, Targeted therapy, Crizotinib, Pazopanib, ALK fusions, Uterine myxoid tumors, Smooth muscle tumor of uncertain malignant potential (STUMP)

## Abstract

**Background:**

Recurrent, metastatic mesenchymal myxoid tumors of the gynecologic tract present a management challenge as there is minimal evidence to guide systemic therapy. Such tumors also present a diagnostic dilemma, as myxoid features are observed in leiomyosarcomas, inflammatory myofibroblastic tumors (IMT), and mesenchymal myxoid tumors. Comprehensive genomic profiling was performed in the course of clinical care on a case of a recurrent, metastatic myxoid uterine malignancy (initially diagnosed as smooth muscle tumor of uncertain malignant potential (STUMP)), to guide identify targeted therapeutic options. To our knowledge, this case represents the first report of clinical response to targeted therapy in a tumor harboring a *DCTN1-ALK* fusion protein.

**Methods:**

Hybridization capture of 315 cancer-related genes plus introns from 28 genes often rearranged or altered in cancer was applied to >50 ng of DNA extracted from this sample and sequenced to high, uniform coverage. Therapy was given in the context of a phase I clinical trial ClinicalTrials.gov Identifier: (NCT01548144).

**Results:**

Immunostains showed diffuse positivity for *ALK1* expression and comprehensive genomic profiling identified an in frame *DCTN1-ALK* gene fusion. The diagnosis of STUMP was revised to that of an IMT with myxoid features. The patient was enrolled in a clinical trial and treated with an anaplastic lymphoma kinase (ALK) inhibitor (crizotinib/Xalkori®) and a multikinase VEGF inhibitor (pazopanib/Votrient®). The patient experienced an ongoing partial response (6+ months) by response evaluation criteria in solid tumors (RECIST) 1.1 criteria.

**Conclusions:**

For myxoid tumors of the gynecologic tract, comprehensive genomic profiling can identify clinical relevant genomic alterations that both direct treatment targeted therapy and help discriminate between similar diagnostic entities.

## Introduction

Myxoid neoplasms of the uterus are a diverse group of soft tissue tumors presenting diagnostic dilemmas for pathologists [1]. The mainstay of treatment for uterine myxoid neoplasms is surgical resection. However, recurrent and metastatic myxoid neoplasms are challenging to manage medically due to scant evidence-based guidance. The combination of a challenging diagnostic proposition and only limited evidence to guide management is a strong rationale for the use of comprehensive genomic profiling (CGP) in an effort to uncover clinically relevant genomic alterations (CRGA) that suggest possible benefit from targeted therapy. Here, we report the case of a patient with a recurrent, metastatic uterine myxoid neoplasm staining diffusely for ALK1 and harboring a *DCTN1-ALK* fusion identified by CGP who has experienced clinical and radiographic improvement with targeted inhibition of anaplastic lymphoma kinase (*ALK*) (crizotibib/Xalkori®) and additional targeted therapy (pazopanib/Votrient®).

## Patients and methods

### Patient selection and clinical assessments

The team reviewed the medical records of a patient who presented to the Department of Investigational Cancer Therapeutics at The University of Texas MD Anderson Cancer Center following an initial diagnosis of a myxoid uterine neoplasm. With minimal standard of care options left, the patient was advised to participate in a clinical trial. Treatment and consent on the investigational trial and data collection were performed in accordance with the guidelines of The University of Texas MD Anderson Cancer Center Institutional Review Board (IRB). Tumor response was determined using response evaluation criteria in solid tumors (RECIST) (version 1.1) by CT scan obtained every 2 cycles post treatment initiation. Clinical evaluation and assessments were performed per protocol.

### Genomic profiling

Comprehensive genomic profiling was performed using the FoundationOne® assay in a Clinical Laboratory Improvement Amendments (CLIA)-certified, CAP-accredited central laboratory (Foundation Medicine, Cambridge, MA, USA). Hybridization capture of 315 cancer-related genes plus introns from 28 genes often rearranged or altered in cancer was applied to >50 ng of DNA extracted from this sample and sequenced to high, uniform coverage. All classes of genomic alterations, including base substitutions, small insertions and deletions (indels), rearrangements, and copy number alterations, were assessed. Clinically relevant genomic alterations (CRGA) were defined as those suggesting benefit from an approved targeted therapy or directing benefit from mechanism-based clinical trials.

## Results and discussion

### Case history

A female in her 50’s Gravida 0, with a long standing history of gynecologic discomfort with history of laparoscopy and hysteroscopy that showed endometriosis and uterine fibroids presented to the clinic with increasing pelvic pressure sensations and significant cramps, symptoms concerning for an abdomino-pelvic neoplasm. At presentation, her disease was described as a 14–16-week sized globular intra-uterine mass and clinically diagnosed as a leiomyoma. Morcellation was performed, and pathologic examination of the formalin fixed paraffin embedded (FFPE) morcellated tissue revealed a myxoid neoplasm, consistent with a smooth muscle tumor of uncertain malignant potential (STUMP). This pathologic diagnosis was done at the outside institution. The patient was subsequently symptomatically monitored for disease progression. Eight months following diagnosis, the patient reported pelvic pain and underwent a bilateral salpingo-oophorectomy, pelvic lymphadenectomy, and omentectomy. Pathologic examination confirmed metastatic myxoid neoplasm within the pelvis right wall, peritoneum, bladder, and peritoneal cul-de-sac. The patient was again monitored, and 7 months later, follow-up imaging identified a 2-cm mass abutting the right external iliac artery. A laparoscopic procedure was performed and confirmed a recurrence of myxoid tumor. The patient was followed for 2 years in which disease subsequently recurred as a lesion in the liver, multiple vaginal tumors, and recurrent tumor over the external iliac artery. These presumed recurrences were biopsied, confirmed as recurrent disease, and resected. A decision was made to investigate systemic treatment, as local management was not effective.

The patient presented to The University of Texas MD Anderson Cancer Center for therapy recommendations. The patient was seen by the gynecological oncologist, sarcoma medical oncologist, and investigational cancer therapeutics consultant at the clinical center for targeted therapy. The natural history of rapid recurrences after initial local management was clearly inconsistent with a typical STUMP. The specimens were requested for pathology confirmation.

**Fig. 1:**
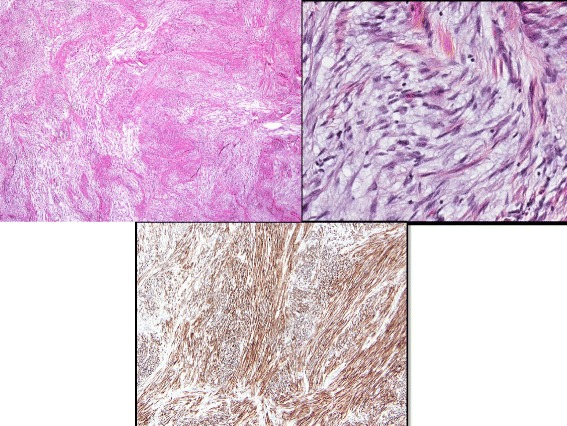
IMT infiltrating the myometrium are images 1 and 2. The myxoid areas are infiltrating the muscle of the myometrium. Image 3 is an immunostain for ALK, diffusely positive in the spindle tumor cells

The diagnostic specimen was immunostained and demonstrated diffuse positivity for ALK as well as positivity for desmin (Fig. 1). At the same time, this specimen was submitted as an FFPE block for comprehensive genomic profiling [2].

### Genomic profiling

**Fig. 2:**
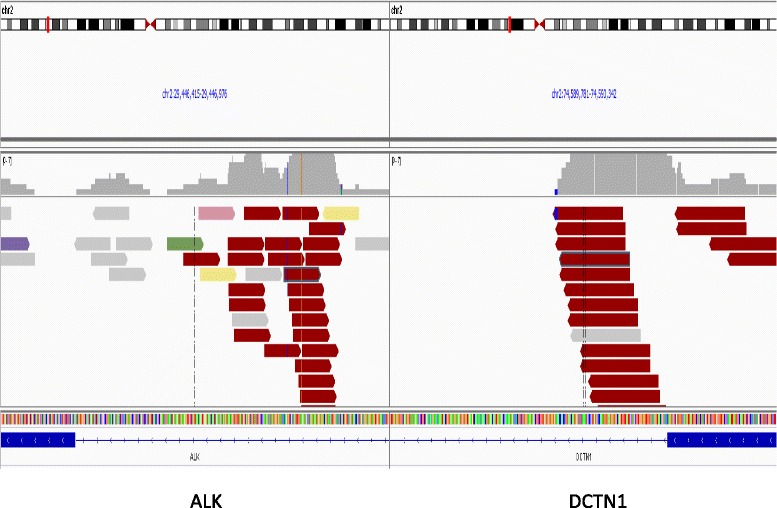
Representative genome images from the Integrated Genome Viewer (IGV) alterations for deletion of ALK exon 1–19, and fusion of ALK-DCTN1 demonstrating ALK and DCTN1 fusion found in the patient with inflammatory myofibroblastic tumor with myxoid features who had a response to ALK inhibitor based therapy

**Fig. 3:**
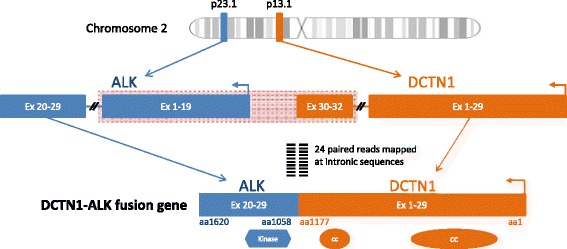
The *DCTN1-ALK* intra-chromosomal rearrangement detected in this case conformed to the common structural organization of ALK fusions, with the vast majority having an ALK intron 19 breakpoint and is a priori suspected to active in vivo. The *DCTN1* component of the fusion contains exons including the coiled-coil domains, which is similar to other previously reported *ALK* fusion partners. Via these domains, *DCTN1* is suspected to promote dimerization of *ALK* and subsequent kinase activation by transphosphorylation

Three hundred fifteen cancer-related genes plus select introns from 28 genes often rearranged or altered in solid tumor cancers were sequenced to a median depth of coverage of 481×. A deletion event on chromosome 2 (Fig. 2), giving rise to a *DCTN1-ALK* fusion (Fig. 3), was identified. No other genomic alterations were identified in the specimen.

Based on the diagnostic results of diffuse ALK expression indicating an alteration of *ALK*, the diagnosis was updated to that of an inflammatory myofibroblastic tumor (IMT) with myxoid features. The comprehensive genomic profiling confirmed the diagnosis by demonstrating a *DCTN1-ALK* fusion.

**Fig. 4:**
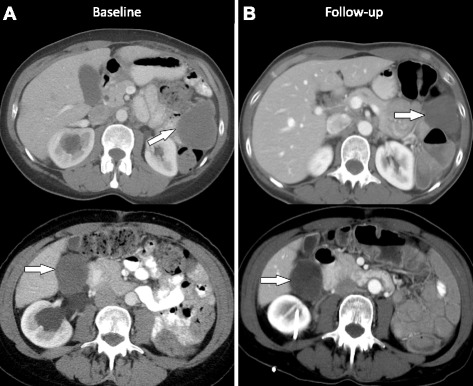
Imaging studies at baseline and follow-up. **a** Pre-treatment axial CT image shows a 6.2 × 5.1 cm peritoneal implant in the left upper quadrant (*top*) and a 4.4 × 4 cm peritoneal implant in the right hepatorenal space (*bottom*). **b** Follow-up axial CT image shows interval decrease in the peritoneal implant in the left upper quadrant which measures 4.5 × 3.8 cm, previously 6.2 × 5.1 cm (*top*). Axial CT image shows that the 4.4 × 4 cm peritoneal implant in the right hepatorenal space (*bottom*) has not changed much in size (measured 4.4 × 3.7 cm), but the lesion has now become less dense. This is an important observation as this represents response to therapy. Radiologists and clinicians should be aware that many targeted therapies may not result in significant change in size particularly during the early stages but may cause interval decrease in vascularity; on CT, this may be seen as decrease in density of the target lesions and represents response to therapy

The patient was presented in the targeted therapy clinical trials treatment planning meeting, and treatment with an ALK inhibitor was recommended. At this time, the patient returned to the Department of Investigational Cancer Therapeutics at The University of Texas MD Anderson Cancer Center for further treatment discussions including a targeted therapy consult for ALK inhibitor options. The patient was enrolled in a phase I clinical trial (ClinicalTrials.gov Identifier: NCT01548144) of crizotinib in combination with pazopanib for the treatment of advanced cancer [3]. The patient was treated with crizotinib 250 mg orally on alternating days and pazopanib 200 mg orally daily for a 21 day cycle. After 2 cycles of therapy, the patient had greater than 30 % reduction in the sum of longest diameter (SLD) of target lesions per RECIST 1.1 (Fig. 4), indicating a partial response to therapy. The patient tolerated the combination therapy well. She had grade 1 mild diarrhea which was controlled by loperamide and grade 1 nausea which was controlled by ondansetron. At the time of submission, the patient continues to have a response for over 6 months with significant decrease in the tumor measurements and is in confirmed partial remission (PR).

Given the revised diagnosis in this case, we wondered if other advanced uterine neoplasms harbored ALK aberrations and rearrangements. A large series of comprehensive genomic profiles of advanced uterine leiomyosarcomas (LMS) obtained in the course of clinical care were analyzed. Of 139 LMS cases, 5 ALK rearrangements were identified for a frequency of 3.6 % (Elvin et al., manuscript in preparation, personal communication).

## Discussion

ALK inhibitors are the standard of care for *ALK*-rearranged non-small cell lung cancer, and the resulting success is the paradigm for precision oncology [4]. This case harbors a *DCTN1-ALK* fusion which has been previously reported [5, 6] but is described here in a myxoid uterine neoplasm for the first time. After multiple recurrences, *ALK* immunohistochemistry (IHC) was performed concurrently with comprehensive genomic profiling (CGP) on the diagnostic specimen. Based on the tumor harboring an *ALK* fusion and the staining for ALK1 by immunohistochemistry, the diagnosis was changed to an inflammatory myofibroblastic tumor (IMT) with myxoid features. Treatment with a combination of crizotinib and pazopanib was initiated in the context of a clinical trial and yielded an ongoing partial response for this case.

*ALK* rearrangements resulting in an activated ALK fusion protein were first identified in 1994 with the observation of nucelophosmin (*NPM1*)-*ALK* in anaplastic large cell lymphoma [7]. More recently, *ALK* rearrangements have been implicated in a variety of tumor types, notably non-small cell lung carcinoma (NSCLC), inflammatory myofibroblastic tumors (IMT), and renal cancer [4]. Particularly in NSCLC, the presence of an ALK fusion predicts response to crizotinib [4]. The rearrangement observed in this case, *DCTN1-ALK*, has been previously reported in a pediatric case of IMT in the soft tissue of the neck [5] and 6/140 atypical Spitz tumors, the latter as identified by CGP [6].

The most common mechanism of *ALK* fusions is a genomic rearrangement involving the *ALK* locus at 2p23 with the common breakpoint in intron 19 resulting in the dissociation of the 3′ exons 20–29 from the 5′ exons 1–19 [4]. The *DCTN1-ALK* intra-chromosomal rearrangement detected in this case conformed to the common structural organization of ALK fusions, with the vast majority having an ALK intron 19 breakpoint, and is a priori suspected to be active in vivo. The *DCTN1* component of the fusion contains exons 1–29 including coiled-coil domains, which is structurally similar to other previously reported *ALK* fusion partners (Figs. 2 and 3). Via these domains, *DCTN1* is suspected to promote dimerization of *ALK* and subsequent kinase activation by transphosphorylation [5, 8].

Myxoid neoplasms of the uterus are defined by myxoid features but can carry a range of diagnoses [9, 10]. Diagnoses of such tumors rely on clinicopathologic observations to formulate an effective differential diagnosis [11]. From the initial differential, myxoid neoplasms are most often classified by their clinical behavior—benign, locally aggressive, or malignant nature—as well as by an imputed histogenetic origin including myxoid leiomyosarcoma, myxoid liposarcoma, and myxofibrosarcoma among others [11].

In particular, the differential diagnosis for a myxoid neoplasm of the uterus must include IMT of the uterus, which are known to have myxoid features. IMTs are characterized by myofibroblastic spindle cells accompanied by a lymphoplasmacytic inflammatory infiltrate [12, 13]. The myxoid morphologic appearance of an IMT can create diagnostic challenges, particularly if pauci-inflammatory, and the differential includes benign myxoid leiomyoma and malignant myxoid and inflammatory leiomyosarcoma [14].

The diagnostic challenge associated with differentiating between uterine myxoid neoplasms and uterine IMT can be resolved through the identification of kinase fusion events. Previous data have demonstrated that approximately 50 % of IMT cases are *ALK* positive [12, 15, 16]. Additional studies have showed that of *ALK* IHC-negative case, 8/11 (73 %) identified alternate kinase fusions, including 2 cases with *ALK* fusions missed by *ALK* IHC and 6 cases harboring either *ROS1* (4) or *PDGFRβ* (2) fusions [13]. The diagnosis of an IMT with uterine origin is further compounded by its rarity and nonspecific symptoms; thus, this diagnosis is commonly overlooked by clinicians [14, 17]. To date, *ALK* positive uterine IMTs have been reported using immunohistochemistry and FISH methods, with previous studies showing 100 % [11, 18] and 87.5 % [19] *ALK* positivity in female genital tract IMTs, respectively. However, such studies are limited by the diagnostic criteria used to identify IMTs. If an IMT is in the differential diagnosis for a uterine neoplasm, CGP can identify genomic rearrangements of non-ALK kinases, which will strongly support the diagnosis of an IMT as seen in IMT of soft tissue [13]. Knowledge of the natural history of IMT, both of uterine origin and other, is scant. From IMTs located in the lung, 3- and 5-year survival were 82 and 74 %, respectively, with 15 % of patients experiencing recurrent disease [20]. No clinical trials for systemic treatment have focused solely on IMTs, but a recent phase I trial for refractory pediatric solid tumors demonstrated three of seven ALK-rearranged IMT patients had partial responses, and another three had stable disease on crizotinib treatment [21].

Crizotinib is an oral small-molecule tyrosine kinase inhibitor that targets the *ALK*, *MET*, and *ROS1* tyrosine kinases and is well known to induce clinically significant responses in NSCLC [22–24]. Response to crizotinib in IMT harboring *ALK* fusions have also been observed in two patients [21, 25, 26]. In this case report, we describe the documented clinical response of a uterine IMT to a combination of crizotinib and pazopanib. The question remains if response, toxicity, or overall survival benefitted from combination therapy of crizotinib and pazopanib compared with crizotinib monotherapy. As exemplified by trials for BRAF V600E metastatic melanoma, treatment with dabrafenib monotherapy versus the combination of dabrafenib and trametinib in BRAF V600E mutated melanoma [27] can significantly improve overall survival without increasing overall toxicities. Crizotinib has been shown to be superior to standard chemotherapy in advanced non-small-cell lung cancer [28] and is approved as the first line therapy for *ALK*-rearranged metastatic NSCLC.

However, acquired resistance to crizotinib monotherapy is well demonstrated in the setting of NSCLC, and a similar phenomenon may arise in *ALK*-rearranged IMT treated with crizotinib [29]. Pazopanib is a multikinase inhibitor with activity against VEGFR1-3, PDGFRA and PDGFRB, FGFR1 and FGFR3, and c-Kit [30]. Whether the combination of crizotinib or pazopanib is synergistic, additive, or deleterious is unknown, and the continuing outcome of the patient may anecdotally address this issue. Arguably, pazopanib is likely to further inhibit signaling downstream of the above receptor tyrosine kinases [30]. This depression of signaling may further benefit the case by inhibiting pathways of acquired resistance akin to PI3K inhibitors and crizotinib in *ALK*-rearranged NSCLC but would need to be explored rigorously in vitro to prove this possibility.

## Conclusions

This report demonstrates the first documented response of a myxoid tumor of the female genital tract harboring an *ALK* fusion to combination therapy including ALK-targeted therapy. To our knowledge, this case represents the first report of clinical response to targeted therapy in a tumor harboring a *DCTN1-ALK* fusion protein. This report not only demonstrates the difficulty in diagnosing rare tumors in uterine soft tissue but also emphasizes the use of comprehensive genomic profiling for identifying clinically relevant genomic alterations, such as ALK rearrangements, and immediate implications for patient benefit.

## Findings

- *DCTN1-ALK* fusion, previously reported in an inflammatory myofibroblastic tumor (IMT) and Spitz tumors, is described here in a myxoid neoplasm of the female reproductive tract.
- This case is the first report of a tumor harboring *DCTN1-ALK* being treated with combination targeted therapy with a documented clinical and radiographic response.
- Comprehensively genomic profiling can uncover clinically relevant genomic alterations in rare tumors, such as myxoid uterine neoplasms, that present diagnostic challenges to pathologic and while simultaneously providing unanticipated pathways to benefit from targeted therapy.

## Consent section

Written informed consent was obtained from the patient for enrolling on the clinical trial and for publication of this case report and any accompanying images. A copy of the written consent is available for review by the Editor-in-Chief of this journal.

## Abbreviations

ALK: anaplastic lymphoma kinase
IMT: inflammatory myofibroblastic tumors
STUMP: smooth muscle tumor of uncertain malignant potential
CGP: comprehensive genomic profiling
CRGA: clinically relevant genomic alterations
CLIA: Clinical Laboratory Improvement Amendments
FFPE: formalin fixed paraffin embedded
IHC: immunohistochemistry
NPM1: nucelophosmin

## References

[1] Zulfiqar MI, Sheikh UN, Montgomery EA (2011). Myxoid neoplasms. Surg Pathol Clin.

[2] Frampton GM, Fitchtenholtz A, Otto GA, Wang K, Downing SR, He J (2013). Development and validation of a clinical cancer genomic profiling test based on massively parallel DNA sequencing. Nat Biotechnol.

[3] Zinner R. Pazopanib or pemetrexed and crizotinib in advanced cancer. (M.D. Anderson Cancer Center). at https://clinicaltrials.gov/ct2/show/NCT01548144.

[4] Marino-Enriquez A, Dal CP (2013). ALK as a paradigm of oncogenic promiscuity: different mechanisms of activation and different fusion partners drive tumors of different lineages. Cancer Genet.

[5] Wang X, Krishnan C, Nguyen EP, Meyer KJ, Oliverira JL, Yang P (2012). Fusion of dynactin 1 to anaplastic lymphoma kinase in inflammatory myofibroblastic tumor. Hum Pathol.

[6] Wiesner T, He J, Yelensky R, Esteve-Puig R, Botton T, Yeh I (2014). Kinase fusions are frequent in Spitz tumors and spitzoid melanomas. Nat Commun.

[7] Morris SW, Kirstein MN, Valentine MB, Dittmer KG, Shapiro DN, Saltman DL (1994). Fusion of a kinase gene, ALK, to a nucleolar protein gene, NPM, in non-Hodgkin’s lymphoma. Science.

[8] Chiarle R, Voena C, Ambrogio C, Piva R, Inghirami G. The anaplastic lymphoma kinase in the pathogenesis of cancer. Nat Rev Cancer. 2008;8. 10.1038/nrc2291 18097461

[9] Allen PW (1980). Myxoid tumors of soft tissues. Pathol Annu.

[10] Mackenzie DH (1981). The myxoid tumors of somatic soft tissues. Am J Surg Pathol.

[11] Graadt van Roggen JF, Hogendoorn PC, Fletcher CD. Myxoid tumours of soft tissue. Histopathology. 1999;35:291–312. 10.1046/j.1365-2559.1999.00835.x 10564384

[12] Coffin CM, Patel A, Perkins S, Elenitoba-Johnson KS, Perlman E, Griffin CA (2001). ALK1 and p80 expression and chromosomal rearrangements involving 2p23 in inflammatory myofibroblastic tumor. Mod Pathol.

[13] Lovly CM, Gupta A, Lipson D, Otto G, Brennan T, Chung CT (2014). Inflammatory myofibroblastic tumors harbor multiple potentially actionable kinase fusions. Cancer Discov.

[14] Fraggetta F, Doglioni C, Scollo P, Pecciarini L, Ippolito M, Amico P, et al. Uterine inflammatory myofibroblastic tumor in a 10-year-old girl presenting as polypoid mass. J Clin Oncol. 2015;33. 10.1200/JCO.2013.48.8304 24590652

[15] Gleason BC, Hornick JL (2008). Inflammatory myofibroblastic tumours: where are we now?. J Clin Pathol.

[16] Cook JR, Dehner LP, Collins MH, Ma Z, Morris SW, Coffin CM (2001). Anaplastic lymphoma kinase (ALK) expression in the inflammatory myofibroblastic tumor: a comparative immunohistochemical study. Am J Surg Pathol.

[17] Kushnir CL, Gerardi M, Banet N, Shih I-M, Diaz-Montes T. Extrauterine inflammatory myofibroblastic tumor: a case report. Gynecol Oncol Case Rep. 2013;6. 10.1016/j.gynor.2013.07.007 PMC3862308 24371717

[18] Parra-Herran C, Quick CM, Howitt BE, Dal Cin P, Quade BJ, Nucci MR (2015). Inflammatory myofibroblastic tumor of the uterus: clinical and pathologic review of 10 cases including a subset with aggressive clinical course. Am J Surg Pathol.

[19] Fuehrer NE, Keeney GL, Ketterling RP, Knudson RA, Bell DA (2012). ALK-1 protein expression and ALK gene rearrangements aid in the diagnosis of inflammatory myofibroblastic tumors of the female genital tract. Arch Pathol Lab Med.

[20] Melloni G, Carretta A, Ciriaco P, Arrigoni G, Fieschi S, Rizzo N (2005). Inflammatory pseudotumor of the lung in adults. Ann Thorac Surg.

[21] Mosse YP, Lim MS, Voss SD, Wilner K, Ruffner K, Laliberte J (2013). Safety and activity of crizotinib for paediatric patients with refractory solid tumours or anaplastic large-cell lymphoma: a Children’s Oncology Group phase 1 consortium study. Lancet Oncol.

[22] Kwak EL, Bang YJ, Camidge DR, Shaw AT, Solomon B, Maki RG (2010). Anaplastic lymphoma kinase inhibition in non-small-cell lung cancer. N Engl J Med.

[23] Ou SH, Kwak EL, Siawak-Tapp C, Dy J, Bergethon K, Clark JW (2011). Activity of crizotinib (PF02341066), a dual mesenchymal-epithelial transition (MET) and anaplastic lymphoma kinase (ALK) inhibitor, in a non-small cell lung cancer patient with de novo MET amplification. J Thorac Oncol.

[24] Bergethon K, Shaw AT, Ou SH, Katayama R, Lovly CM, McDonald NT (2012). ROS1 rearrangements define a unique molecular class of lung cancers. J Clin Oncol.

[25] Butrynski JE, D’Adamo DR, Hornick JL, Dal Cin P, Antonescu CR, Jhanwar SC (2010). Crizotinib in ALK-rearranged inflammatory myofibroblastic tumor. N Engl J Med.

[26] Lorenzi L, Cigognetti M, Medicina D, Pellegrini V, Balzarini P, Cestari R (2014). ALK-positive inflammatory myofibroblastic tumor of the abdomen with widespread microscopic multifocality. Int J Surg Pathol.

[27] Robert C, Karaszewska B, Schachter J, Rutkowski P, Mackiewicz A, Stroiakovski D (2015). Improved overall survival in melanoma with combined dabrafenib and trametinib. N Engl J Med.

[28] Shaw AT, Kim DW, Nakagawa K, Seto T, Crino L, Ahn MJ (2013). Crizotinib versus chemotherapy in advanced ALK-positive lung cancer. N Engl J Med.

[29] Stinchcombe, T. E. Recent advances in the treatment of non-small cell and small cell lung cancer. F1000Prime Rep. 2014;6:117. 10.12703/P6-117 PMC4251418 25580271

[30] Van Geel RM, Beijnen JH, Schellens JH (2012). Concise drug review: pazopanib and axitinib. Oncologist.

